# Untargeted metabolomics for uncovering biological markers of human skeletal muscle ageing

**DOI:** 10.18632/aging.103513

**Published:** 2020-06-24

**Authors:** Daniel J. Wilkinson, Giovanny Rodriguez-Blanco, Warwick B. Dunn, Bethan E. Phillips, John P. Williams, Paul L. Greenhaff, Kenneth Smith, Iain J. Gallagher, Philip J. Atherton

**Affiliations:** 1 MRC-Versus Arthritis Centre for Musculoskeletal Ageing Research, University of Nottingham, Nottingham, UK; 2 National Institute for Health Research (NIHR) Nottingham Biomedical Research Centre, Nottingham, UK; 3 School of Medicine, University of Nottingham, Royal Derby Hospital Centre, Derby, UK; 4 School of Life Sciences, University of Nottingham, Queens Medical Centre, Nottingham, UK; 5 School of Biosciences and Phenome Centre Birmingham, University of Birmingham, Birmingham, Birmingham, UK; 6 University of Stirling, Faculty of Health Sciences and Sport, Stirling, UK; 7 Beatson Institute for Cancer Research, Glasgow, UK

**Keywords:** metabolomics, muscle, aging, markers

## Abstract

Ageing compromises skeletal muscle mass and function through poorly defined molecular aetiology. Here we have used untargeted metabolomics using UHPLC-MS to profile muscle tissue from young (*n*=10, 25±4y), middle aged (*n*=18, 50±4y) and older (*n*=18, 70±3y) men and women (50:50). Random Forest was used to prioritise metabolite features most informative in stratifying older age, with potential biological context examined using the prize-collecting Steiner forest algorithm embedded in the PIUMet software, to identify metabolic pathways likely perturbed in ageing. This approach was able to filter a large dataset of several thousand metabolites down to subnetworks of age important metabolites. Identified networks included the common age-associated metabolites such as androgens, (poly)amines/amino acids and lipid metabolites, in addition to some potentially novel ageing related markers such as dihydrothymine and imidazolone-5-proprionic acid. The present study reveals that this approach is a potentially useful tool to identify processes underlying human tissue ageing, and could therefore be utilised in future studies to investigate the links between age predictive metabolites and common biomarkers linked to health and disease across age.

## INTRODUCTION

The combined loss of both skeletal muscle mass (sarcopenia: [1]) and strength (dynapenia: [2–4]) in ageing, not only impairs locomotory function, impacting independence and quality of life, but can also compromise metabolic health. Reflecting this, there are robust epidemiological relationships between both sarcopenia and dynapenia, risk of non-communicable diseases (e.g. diabetes, COPD, cancer) and subsequent morbidity and mortality [5–10]. Identifying biological signatures associated with muscle ageing status is a potential approach to triage those in need of early intervention, develop tools for stratification or reveal targets for therapeutic manipulation [11–13].

In recent years there has been increased use of OMICs-technologies (e.g. transcriptomics, proteomics and metabolomics) to examine the underlying aetiology of disease states and develop biomarkers for diagnosis or prognosis [14–16]. Metabolomic approaches, for instance, have already yielded insight into changes in the muscle metabolome with age. Fazelzadeh and colleagues recently used a targeted approach to investigate age-related changes in the muscle metabolome and highlighted that metabolites associated with mitochondrial function, fibre type and tissue turnover all differed between age groups [17], in-keeping with established ageing physiology [18–20]. Johnson and colleagues examined the relationship between metabolites and indicators of health span, and demonstrated that blood concentrations of certain amino acids and lipids were associated with health-span indicators in ageing [21]. What is still not known, is whether changes in the skeletal muscle metabolome associated with ageing could be informative of an ageing tissue phenotype and link to other clinical health-related parameters. However one of the major issues in metabolomics is determining the most optimal approach for identification of these “biologically important” changes in the metabolome associated with ageing, so that these links to clinical/health outcomes can be determined and investigated further.

Untargeted metabolomics datasets are notoriously difficult to analyse. Firstly, there is the ‘curse of dimensionality’ whereby the number of observed variables is much larger than the number of samples used to observe those variables. This leads to a large false positive rate using significance based statistical approaches. Secondly the ‘dark matter’ of untargeted metabolomics datasets means that physiologically principled approaches to variable importance are difficult because many metabolites cannot be identified [22]. Machine learning algorithms such as Random Forest (RF: [23]) can be used to identify potentially informative variables in such datasets [24]. RF is well suited to high dimensional datasets for several reasons; RF is non-parametric, difficult to over-train, robust to outliers, provides information on variable importance and also provides built-in cross-validation [25]. These properties make RF a useful choice for metabolite prioritization in untargeted metabolomic studies. Another layer of support can be added by providing evidence of an ‘expected’ biological context. We believe this represents a pragmatic approach to analysis of a high-dimensional dataset where variable selection by physiological consideration would fail (because many metabolites cannot be identified), assumptions (e.g. independence of metabolites) for approaches like ANOVA are likely violated and traditional statistical power is difficult to achieve or even define due to high-dimensionality of the data.

Here we use the approach outlined above. First we use the RF algorithm to screen an untargeted metabolomics dataset and identify metabolites potentially informing on chronological ageing in muscle tissue. Then we used the PIUMet software which implements the prize-collecting Steiner Forest algorithm to identify putative metabolic networks containing these metabolites which may be perturbed in older muscle and assess the biological context of the network members.

## RESULTS

### Random forest selection of metabolite features of predictive importance that stratify human muscle ageing

The impact of metabolite selection using RF is seen in Figure 1. Prior to application of RF there is a similar level of variability in samples across the age groups, and no distinct clustering of the groups (Figure 1A). After application of RF we were able to identify metabolites that can separate the full cohort age class (Figure 1B, 1C) and plotting principle components demonstrates a gradient effect across age groups in PC1 (old to young over PC1) (Figure 1D). This is the pattern we would expect to see if these metabolites are informative on age across the age-span of the study. The arrows in Figure 1D show loadings which represent the weight of the labelled variables in the direction of older age. Thus RF was able to select potentially informative metabolites for further analysis. Figure 1E shows representative boxplots for potentially informative metabolite levels in the polar positive modality. The abundance of each metabolite across the classes reflects the direction of the arrows in the biplot (see Figure 1D) highlighting the potential differences between age groups for these metabolite features. Clear differences observed between metabolite abundance for each age group. Although not our primary objective we also examined the ability of RF to classify subjects according to age group. RF performed well when predicting older age (OOB error for old between 0% (RP neg) and 11% (HILIC Pos)) when using the young as a reference group, due to younger age being harder to classify using RF (OOB ranging from 50-100% for young)

**Figure 1:**
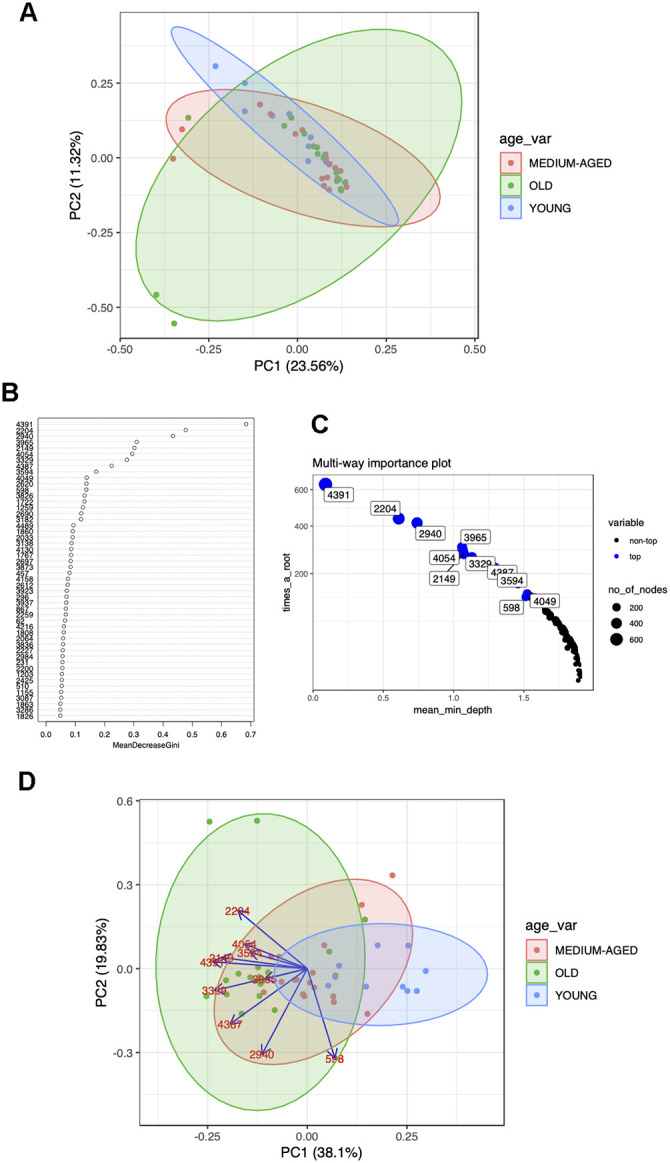
(**A**) Pre RF PCA plot showing overlap of age groups and no defined clusters of metabolites predictive of age group for polar positive data. (**B**) Example variable importance and (**C**) multi-way importance plots generated from RF for polar positive data and the use of the randomForestExplainer R package. The most important predictive metabolites are selected out via Gini index and the top 10 (although arbitrary, this is generally selected as to where the variable importance falls off, ie as shown in the plot of panel **B**) selected for each polarity and ion mode to go forward for further analyses. (**D**) Post RF PCA plot for polar positive data, reduction of data to those metabolites most predictive of age shows clustering of age groups with most variability between age groups contained in PC1, with the direction and degree of correlation between each metabolite driving this difference shown through the loadings.

**Figure 1:**
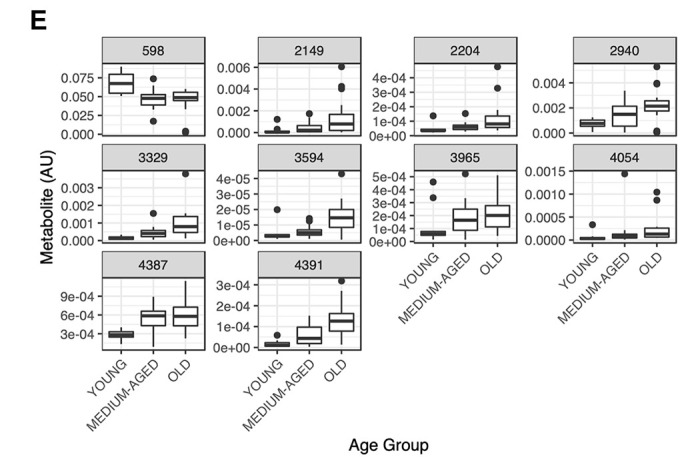
(**E**) Representative boxplots for RF selected metabolites showing differences in metabolite abundance across age groups for these variables.

### Annotation and identification of selected age predictive metabolite features

Of the 42 metabolites selected via RF (10 per polarity and modality, except RP Pos which provided 12 due to equal importance scores), putative ID’s were found for 31 using the PIUMet algorithm. Due to the difficulty associated with metabolite annotation in untargeted metabolomics [26], it was not possible to uniquely identify all metabolite features within a given mass tolerance (5ppm; see Supplementary Table 1 for full list of ID matches).

**Figure 2:**
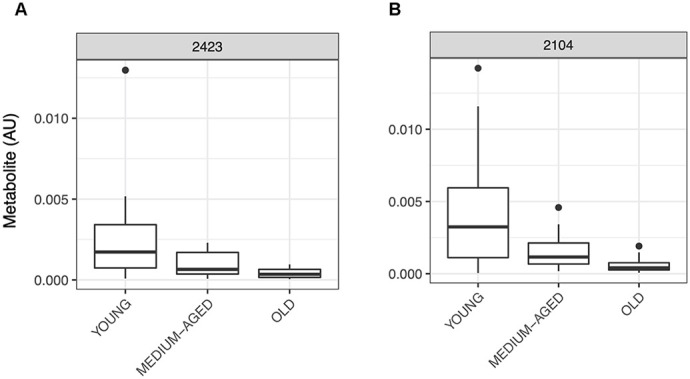
**Representative boxplots of metabolite abundance for metabolites A) 2423 and B) 2104 which were selected via RF to be predictive of muscle age and were matched to a number of steroid and androgen metabolites following annotation using the PIUMet algorithm.** Relative abundance for both metabolite features shows decline with age, a well-established relationship observed in ageing muscle for steroid and androgen metabolites.

A number of metabolite matches provide plausible, biologically informed support for our approach and provide confidence for the use of RF in selecting metabolites important for informing on processes associated with aging. For example, metabolite 2423 (MS predicted mw: 370.18135) (Figure 2A) matched a number of metabolites including Androsterone Sulfate and 5a-Dihydrotestosterone Sulfate, while metabolite 2104 (MS predicted mw: 368.16575) (Figure 2B) matched to metabolites including Epitestosterone Sulfate, Dehydroepiandrosterone Sulfate and Testosterone Sulfate, which all relate to androgen steroid metabolism. It is well established that androgens such as testosterone decline with age in both men and women, and that this is associated with both declines in muscle mass and function [27]. Therefore, it may be expected that alterations in testosterone metabolism would be indicative of muscle ageing, and through the close association of testosterone levels with muscle mass, androgen metabolites could be predictive of human muscle ageing. Metabolite abundance in skeletal muscle for these two androgen related metabolites show declines across the age groups, which is line with previous literature ([28]: Figure 2A, 2B)

A number of other groups were identified by PIUMet from the metabolites predictive of age, including lipid-based metabolites (Lysophospholipids, itaconic acid, capryloylglycine), amines (spermine, histamine), amino acid metabolites (histamine, imadazolone-5-propionic acid) and energy metabolites (Phosphocreatine). All of these increased in abundance with age.

### Ageing-muscle metabolome networks

**Figure 3:**
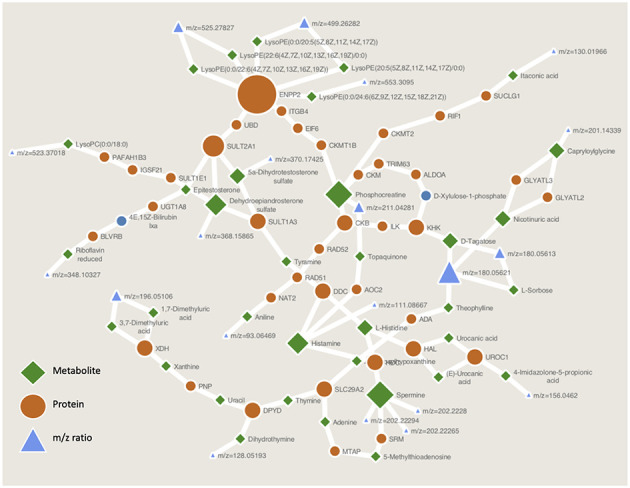
**Metabolite network built through PIUMet.** Key metabolite subnetworks centred around histamine, androgen and phospholipid metabolism, and phosphocreatine are highlighted as hubs for this ageing network.

After metabolite identification (Supplementary Table 1), the PIUMet algorithm generates a metabolite network using the prize-collecting Steiner Forest algorithm, and identifies a subset of metabolites most likely to correspond to the matching metabolite feature, providing a robustness score for each in relation to the network parameters (see Pirhaji et al. [29] for more details). Using this method, we generated a metabolite network associated with human muscle ageing (Figure 3). This network highlights proposed links between identified metabolites including potential protein-metabolite and protein-protein interactions. Notably, subnetworks were generated around physiologically relevant hubs such as phosphocreatine, androgen metabolism, histamine and lysophospholipid metabolism (see Figure 3). These data indicate a potential role for these metabolites, or the subnetworks involved with these metabolites, in human muscle ageing.

## DISCUSSION

In the present study we used the RF algorithm to prioritise metabolites informative for age group from a large dataset of several thousand metabolites. We then used the PIUMet algorithm to identify metabolites and generate a metabolite network structure that reflected the known biology of human ageing and effects on muscle. This network included age associated metabolites such as phosphocreatine, androgens, amines/amino acids (histamine, histidine) and lipid metabolites as well as novel ageing related markers such as dihydrothymine, a marker of DNA damage and imidazolone-5-proprionic acid.

Changes in Androgen metabolism were apparent in the ageing muscle metabolite signature, with the metabolites dehydroepiandrosterone sulfate and 5-alpha-dihydrotestosterone sulfate showing declines in abundance with increasing age (see Figure 2A, 2B). The association between testosterone and ageing is long established; both total and free testosterone decline with age in men and women [29]. Moreover, age-related declines in testosterone have been linked to reduced muscle mass and strength [30], immobility, physical performance [31], and frailty [27]. Further substantiating our findings, in a recent untargeted metabolomics study, it was demonstrated that chronological age was significantly correlated with the steroid/androgen metabolites 4-androsten-3beta, 17beta-dioldmonosulfate and 4-androsten-3beta, 17beta-diol disulfate 2 [32]. The present findings therefore provide good confidence that our RF based bioinformatics workflow is able to yield useful information about the metabolomic signature of ageing skeletal muscle.

The lipid composition of the muscle cell influences membrane structure, function, permeability, molecular transport, intracellular signalling [33] and the synthesis of steroid hormones including the androgens [34]. This may explain the link between lipid-based metabolites and androgens in our PIUMet predicted network. With ageing, the composition of lipids shifts towards saturated intramyocellular lipids [35, 36] which when coupled to declines in mitochondrial content [37], can lead to insulin resistance [38]. In support of these changes to lipid composition with age, we report that a number of lipid compounds were identified in our bioinformatic analysis and changed with age; namely the Lysophospholipids (LysoPE(0:0/22:6)/(22:6/0:0), LysoPE(0:0/20:5)/(20:5/0:0), LysoPE(0:0/24:6) and LysoPC(0:0/18:0), the branched fatty acid Itaconic Acid and the medium chain acylglycine Capryloylglycine. The lysophospholipids are produced via the breakdown of membrane phospholipids [39]. The apparent functional relationship between increases in lysophophospholipids in the present study and breakdown of cellular membranes may have links to musculoskeletal ageing e.g. with a decrease in muscle mitochondrial phosphatidylethanolamine being observed in ageing mice [40]. This decline in mitochondrial phosphatidylethanolamine may be implicit in the declines in mitochondrial function observed with ageing [41], and associated functional decline in muscle. Further, the lysophospholipids have important intracellular signalling roles regulating processes as diverse as blood pressure regulation, cell proliferation, cell survival and cell morphology [42, 43]; in particular lysophosphatidic acid (LysoPA) and lysophosphatidylcholine (LysoPC). Recent findings have also linked the levels of LysoPC in plasma to chronological ageing [44], which we now add support to, albeit in muscle, within the present study i.e. increased levels in both of the LysoPC and LysoPE species. Such changes in abundance of these compounds may contribute to age-associated muscle decline and require follow-up.

Itaconic acid or Itaconate is a small branched-chain fatty acid, originally identified as compound produced by the fungus Aspergillus [45]. Recently this compound has been found to be produced by macrophages [46] linked to the M1 polarization phase of the macrophage response to pro-inflammatory signals in order to remove foreign and damaged cells [47]. This M1 polarization leads to impairments to the flux through the TCA cycle, with Itaconic acid being formed by the decarboxylation of the TCA intermediate cis-acotinate through the enzyme immune-responsive gene (Irg1; [48]). Itaconate can then act as an antimicrobial agent by disrupting the glyoxylate cycle used by pathogens [47, 49]. Ageing has long been associated with inflammation, and associated declines in skeletal muscle mass and function [50], as well as impaired immune responsiveness [51, 52]. Therefore, increases in itaconate with age, may relate to an increased inflammatory immune response; indeed, there is evidence in rats that itaconate is capable of reducing levels of visceral fat through the inhibition of F6P2Kinase [45], potentially responding on two fronts to the ageing inflammatory response.

Capryloylglycine or n-octanoylglycine is a medium chain fatty acid. It is produced as a result of acylCoA esters conjugating with glycine through the enzyme glycine N-acyltransferase [53], and is normally observed in urine as a marker of in-born errors of metabolism such as medium chain acyl CoA dehydrogenase deficiency (MCADD; [54]). The process leading to the increase in capryloylglycine with age in the present study is unclear. Yet, with its role in regulation of acylCoA levels, it may relate to impaired mitochondrial function or content with age. With many of the lipid metabolites identified providing the potential for impairment to energetic pathways, particularly involving the mitochondria and oxidative phosphorylation, it may be expected that other aspects of energy metabolism are perturbed in ageing muscle. In support of this thesis Phosphocreatine was also identified as a key metabolite in ageing muscle in the present findings, with the PIUMet predictive network linking it to both Itaconic acid and LysoPE (see Figure 3)

The polyamines; Spermine and Spermidine have been compounds of continued interest in ageing and health research in recent years [55]. At a systems level they are considered essential growth factors for cells, being involved in multiple processes from signal transduction to protein and DNA synthesis, and have key roles in the regulation of skeletal muscle mass [56]. Here we found spermine to be predictive of age, with increased abundance with age (not significant following FDR correction), which supports previous muscle metabolome data looking at differences between old and young cohorts [17]. Reduced expression of spermine oxidase (SMOX), leading to increased spermine levels, has been shown to be linked with muscle atrophy in mouse models [57]. Concomitantly overexpression of SMOX in C2C12 murine myotubes increases overall fibre size [58]. Moreover, in a number of neuromuscular disorders, an increased concentration of the polyamines and associated metabolites is often observed [59, 60], highlighting their vital role in the skeletal muscle mass regulation, and indicating that they might be related to impaired regulation of muscle mass with ageing.

The synthesis and degradation of polyamines is closely linked to the metabolism of another amine identified within our metabolite signature of muscle ageing - histamine. The interplay between the two amine pathways is still not fully understood, however histamine may impact expression of the enzyme ornithine decarboxylase (ODC), a key step in polyamine synthesis [61–63]. Histamine is a prominent vasoactive substance, helping regulate blood-flow and vascular tone [64, 65]. It is well established that the control of blood flow is impaired in ageing with significant decrease in flow and microvascular perfusion to the muscle in response to nutritive stimuli [66, 67]. Though speculative, increased levels of histamine observed in older muscle could represent a compensatory mechanism. Interestingly, links between amines, polyamines and androgens exist, hence the predicted links between these compounds in the PIUMet network analyses (Figure 3). For instance, treatment of orchidectomized male mice with testosterone led to increased expression of ODC, regulating polyamine synthesis and degradation [68, 69]. Furthermore androgen receptor knockout leads to the down regulation in expression of ODC and other enzymatic proteins, in muscle, involved in polyamine biosynthesis [56]. These interlinked pathways could be implicit in the regulation of skeletal muscle mass with ageing and could provide useful therapeutic targets for future investigation.

In addition to those metabolites which formed the major hubs and subnetworks for the present ageing muscle signature, there were a small number of other minor metabolites which were also shown to be powerful in age prediction, namely imadozolone-5-proprionic acid, dihydrothymine, 1,7/3,7 dimethyluric acid, aniline and tagatose. Imadozolone-5-proprionic acid is a metabolite of histidine closely linked to histamine within the predicted network via histidine and the enzyme histidine decarboxylase, which catalyses the decarboxylation of histidine to histamine [70]. Dihydrothymine is a breakdown product of the nucleotide thymine, however it is also believed to represent the presence of potential DNA damage [71]. The idea that DNA damage may be a contributing factor towards cellular ageing has long been proposed [72, 73], and therefore could indicate the presence of DNA damage in ageing muscle. 1,7/3,7-dimethylurate is another metabolite of nucleotide metabolism associated with the purine xanthine. It is also known as a breakdown product and marker of the pharmacokinetics of the COPD and asthma drug Theophylline or Dimethylxanthine [74], and in the metabolism of caffeine [75]. Interestingly the isomer 1,7 dimethylurate in plasma has been shown to be related to chronological ageing in previous untargeted metabolomics studies [32]. This provides support for this compound as a potentially novel marker, with more research needed into its potential biological actions and putative links to ageing. Both aniline and tagatose were putatively identified within these samples. Tagatose is common in sweeteners in food and dairy products and therefore may be reflective of dietary differences, whereas aniline is an industrial chemical not expected to be present endogenously and therefore may reflect a contaminant or an inaccurately identified compound.

This work is primarily exploratory in nature and is not intended to define a definitive metabolic signature of ageing and health. It is more an attempt to generate leads and hypotheses to investigate in future, i.e. through identification of metabolites and/or metabolic pathways for manipulation via pharmaceutical and drug targets in models of ageing and disease using a more targeted approach, and to assist in confirming a potential ageing metabolite signature and associated therapeutic intervention for health across the lifespan. Moreover, it is also important to consider the aspect of gender in the context of future work, whilst not possible in the current study, there is clear evidence of sexual dimorphism in the context of skeletal muscle aging [76]. It is therefore possible that metabolite signatures may show distinct gender specific differences particularly in those associated with androgen metabolism. In addition to this, the sample size could be considered somewhat limited in the current study when it comes to OMICs, and therefore statistical power could be compromised in this type of analyses. However, the use of RF is optimal for dealing with so called “small n large p” data sets [77] by minimising the potential for overfitting data. However, follow up work with a larger cohort is clearly needed to independently validate our findings. A current major problem in the field of metabolomics, are the issues involved with metabolite feature annotation and accuracy of ID in untargeted metabolomics [26]. Despite significant progress in recent years, annotation is still lagging far behind the other OMICs approaches such as proteomics and genomics/transcriptomics.

The present study reveals that a novel bioinformatics-based metabolomics approach involving the use of both Random Forest to detect metabolites important to predict age and PIUMet to fit these to predicted metabolic networks, is a potentially useful tool to classify human tissue ageing. This process provides a pragmatic approach to datasets such as those seen in untargeted metabolomics where many potentially biologically informative entities cannot be identified, and false positive rates are potentially very high. Based on the work presented here, future untargeted work through recruitment of a second independent cohort would be needed to validate this approach and the identification of the potential age important metabolites presented here, this could then be followed by a more robust targeted validation in a third independent cohort. Once fully validated this approach could be utilised in future studies to investigate the links between age predictive metabolites and common biomarkers linked to health and disease across our ageing population, and therefore could be used to assist towards the identification of novel preventative measures for age associated diseases.

## MATERIALS AND METHODS

### Subject recruitment, ethics and study data collection

This work utilises samples collected as part of previously published work [78]. This study was reviewed and approved by the University of Nottingham Medical School Ethics Committee (D/2/2006) and was performed in accordance with the Declaration of Helsinki. All subjects gave written informed consent to participate in the study prior to inclusion after all procedures and risks were explained.

Three groups of subjects consisting of young (*n* = 10, 25 ± 4 yr; BMI 24 ± 1 kg/m^2^), middle-aged (*n* = 18, 50 ± 4 yr; BMI 27 ± 1 kg/m^2^), and older (*n* = 18, 70 ± 3 yr; BMI 27 ± 1 kg/m^2^) men and women (~50:50) who were well matched for baseline lean mass, were recruited (see Table 1 for summary of subject characteristics). All subjects were screened by means of a medical questionnaire, physical examination, and resting ECG, with exclusions for moderate muscle wasting (>1 SD below age norms); metabolic, respiratory, or cardiovascular disorders; or other signs and symptoms of ill health. Once enrolled in the study, volunteers were instructed to refrain from exercise for 72 hours and return to the laboratories for testing in an overnight fasted state. Upon arrival, body composition was measured using dual-energy X-ray absorptiometry. Volunteers then provided a venous blood sample for the measurement of fasting insulin, glucose, cholesterol and triglycerides, followed by measurements of resting heart rate (RHR) and mean arterial pressure (MAP). Muscle biopsies of the *m*. *vastus lateralis* were then taken under sterile conditions using the conchotome biopsy technique [79], with 1% lidocaine (B. Braun Melsungen) as local anaesthetic. Muscle was rapidly dissected free of fat and connective tissue, washed in ice-cold saline, and snap frozen in liquid N_2_ before storage at –80°C until further analysis.

**Table 1:** Summary of subject characteristics. All data as mean ± SEM.

	**Young**	**Middle aged**	**Old**
**Leg (dominant) Lean Mass (g)**	8510.1 ± 508.3	7975.4 ± 487.9	8160.5 ± 523.3
**Whole Body Strength (N)**	4404.7 ± 308.4	4182.3 ± 357	3570.6 ± 188.7
**% Body Fat**	28.1 ± 4.0	34.5 ± 1.6	32.8 ± 0.2
**Fasting Insulin (μU/ml)**	4.5 ± 0.6	4 ± 0.4	4.9 ± 0.5
**Fasting Glucose (mM)**	5.1 ± 0.1	5.5 ± 0.2	5.8 ± 0.1
**HDL Cholesterol (mM)**	1.2 ± 0.1	1.4 ± 0.1	1.2 ± 0.1
**LDL Cholesterol (mM)**	2.4 ± 0.5	3.1 ± 0.3	3.2 ± 0.2
**Triglycerides (mM)**	0.9 ± 0.1	1.0 ± 0.1	1.1 ± 0.1

### Untargeted metabolomic analysis

### Sample preparation

Approximately 30 mg of muscle tissue was mixed with 1000 μL of methanol:water:chloroform (2.5:1:1 [v/v/v]) and homogenised in a Qiagen TissueLyser II (Qiagen, Germany) at 30 Hz for 2 × 30 s cycle followed by shaking at room temperature for 10 min. Samples were centrifuged (10,000 g, 3 °C, 5 min) followed by transfer of ~1000 μL of the supernatant to a clean 2 mL microcentrifuge tube. 500 μL of HPLC grade water was added followed by vortex mixing and centrifugation (10,000 g, 3 °C, 5 min) to induce phase separation. The upper polar phase (methanol/water) and the lower non-polar phase (chloroform) were transferred in to separate clean 2 mL autosampler vials and dried under nitrogen. Samples were stored at −80 °C until analysis.

### UHPLC-MS

All samples were analysed as described previously [80] using an UltiMate U3000 RSLC UHPLC system coupled to an electrospray Q-Exactive mass spectrometer. The polar phase samples were analysed applying HILIC-MS after being reconstituted in 100 μL of 95/5 acetonitrile/water, and the non-polar phase samples were analysed applying reversed phase C_18_-MS after being reconstituted in 100 μL of 50/50 water/methanol. After reconstitution, 20 μL of each sample was pooled in to a QC sample to quantify technical reproducibility. For HILIC MS, an Accucore 150-Amide HILIC UHPLC column (100 mm × 2.1 mm 2.6 μm, Thermo-Fisher Ltd., UK) was used with mobile phase A: 95% acetonitrile and 5 mM Ammonium Formate (pH 3), and mobile phase B: 5 mM Ammonium Formate in water at (pH 3). For reversed phase MS a Hypersil Gold UHPLC C_18_ column (100 mm × 2.1 mm 1.9 μm, Thermo-Fisher Ltd.) was used with mobile phase A: water with 0.1% formic acid, and mobile phase B: methanol with 0.1% formic acid. 5μL of each sample was injected and analysed applying positive-negative ion mode switching with data collected in the m/z range 100–1000. Ten QC samples were analysed at the start of the analysis followed by a QC sample after every 6^th^ biological sample and two QC samples at the end of the analytical run. Biological samples were randomised across the analytical batch. Gradient elution profiles and mass spectrometer conditions used for each mode are provided in Table 2.

**Table 2A:** UHPLC gradient elution profiles for each polarity.

**HILIC Phase**		
**Time (mins)**	**% Buffer Composition**	
	**Buffer A - Acetonitrile:Water (95:5) with 5mM Ammonium Formate (pH 3)**	**Buffer B - Water:Acetonitrile (95:5) with 5mM Ammonium Formate (pH 3)**
0	95	5
1	95	5
12	55	45
15	55	45
16	95	5
21	95	5
**Reversed Phase (C18)**		
**Time (mins)**	**% Buffer Composition**	
	**Buffer A - Water with 0.1% Formic Acid**	**Buffer B - Methanol with 0.1% Formic Acid**
0	95	5
2	95	5
9	5	95
12	5	95
13	95	5
16	95	5

**Table 2B:** MS operating conditions for each ion mode.

**MS Conditions - Q-Exactive MS**	
Spray Voltage (kV)	3.5 (ESI-)/4.5 (ESI+)
Sheath Gas (AU)	40
Aux Gas (AU)	15
Sweep Gas (AU)	0
S-Lens	100
Resolution	35,000 (FWHM, m/z 200)
Capillary Temp (°C)	300
ESI Heater Temp (°C)	300

### Sample pre-processing

Raw data (.RAW) files were pre-processed using vendor software Compound Discoverer (Thermo Scientific, Bremen, Germany; mass tolerance: 5ppm, Signal/Noise: 3, min number of isotopes: 1) to extract out metabolite features and corresponding accurate mass molecular weight. Data were exported as a data matrix of metabolite feature (molecular weight-retention time pair) vs. sample with associated chromatographic peak areas for a detected metabolite for each polarity (Polar/Non Polar) and ion mode (Positive and Negative). Each metabolite feature with a relative standard deviation calculated for QC samples greater than 20% and not detected in greater than 70% of QC samples were removed prior to further downstream data analysis. This is a commonly applied technique for untargeted metabolomics [81] that has been recommended in recent guidelines [82]. In total, 5655 metabolite features were detected in the muscle samples; 2819 polar positive, 1251 polar negative, 1239 non-polar positive and 346 non-polar negative.

### Statistics

Data for each polarity and ion mode was analysed separately utilising in-house R (R Core Team 2013) scripts. Prior to analysis, and to account for any differences in the amount of muscle processed for each sample (exactly 30mg was not possible for some samples due to low sample amount), SUM normalisation of metabolite abundances was performed [83, 84].

### Random forest selection of metabolite features of predictive importance

The Random Forest (RF) algorithm was used to identify metabolites informative on age class [23]. Intuitively high correlation between predictor features (ill conditioning) means that many features provide the same/similar information. Consequently estimates of e.g. feature importance are diluted. Toloşi and Lengauer explored this issue in RF and other feature selection methods concluding that even very relevant features can be assigned small importance measures if they are highly correlated with many other features [85]. Due to the exploratory nature of our study we took a pragmatic approach and removed highly correlated features (Pearson correlation ≥ 0.9) before applying RF. Features were ranked by the Gini coefficient, a measure of terminal leaf purity for RF trees. With limited numbers of subjects in each group, RF variable selection was performed only on the extremes of age, i.e. old versus young with the expectation that middle aged would fall between these two.

The top 10 most important metabolite features by Gini coefficient from each polarity and ion mode were selected and a reduced data set based on these metabolites was subjected to further analyses. Lists of polar and non-polar metabolites were generated alongside their respective molecular weights, m/z values and predicted chemical formulae following referencing back to pre-processing through Compound Discoverer. For each metabolite feature univariate analyses was performed to detect differences in relative metabolite abundance between old and young using the univariate function (Mann-Whitney U Test) provided in the muma R package [86] and negative log10 of the Benjamini-Hochberg corrected p-values were obtained.

### Metabolite annotation, identification and predicted integrated metabolite network analyses

Lists of metabolite features (identified by their m/z value and polarity) and the results of the univariate test used to determine differences between samples were input into the PIUMet algorithm [87]. PIUMet uses this information and implements a machine learning approach to infer pathways and experimentally undetected components from the list of untargeted metabolite features provided, by utilising an integrative network of over one million protein and metabolite interactions, obtained from the iRefIndex, HMDB and Recon 2 databases (see [87] for more detailed information on PIUMet). Application of PIUMet allowed us to obtain putative metabolite IDs and generate potential molecular/metabolite networks which may be important in the muscle ageing phenotype. The network data was extracted from the PIUMet edge frequency file and the PIUMet network reconstituted in Cytoscape 3.7 [88] for legibility.

## Supplementary Material

Supplementary Table 1
