# Supplementary material for: Untargeted metabolomics for uncovering biological markers of human skeletal muscle ageing
Source: Aging (Albany NY). 2020 Jun 24;12(13):12517–33. doi: 10.18632/aging.103513 (PMC7377844; doi:10.18632/aging.103513)
Supplement: Supplementary Table 1 [file aging-12-103513-s001..docx]

**Supplementary Table 1. Putative Metabolite IDs for Age Important Metabolites Identified by RF.**

| **Study ID Code** | **Metabolite Name** | **Monoisotopic molecular weight** | **Chemical formula** | **Compound Class** | **Compound Origin** | **Cellular Location** | **HMDB ID** | **PIUMet ID** |
| --- | --- | --- | --- | --- | --- | --- | --- | --- |
| **2104** | Epitestosterone sulfate | 368.165745 | C19H28O5S | Organic compounds | Endogenous\|Food | Cytoplasm\|Extracellular\|Membrane | HMDB0013230 | met61611 |
|  | (2S)-2-Butanol O-[b-D-Apiofuranosyl-(1-&gt;6)-b-D-glucopyranoside] | 368.168247 | C15H28O10 | Organic compounds | Food | Cytoplasm\|Extracellular | HMDB0041255 | met72964 |
|  | Kanzonol P | 368.162374 | C22H24O5 | Organic compounds | Food | Membrane | HMDB0041193 | met73028 |
|  | 9-Hydroxy-4-(3,7-dimethyl-2,6-octadienyloxy)-psoralen | 368.162374 | C22H24O5 | Phenylpropanoids and polyketides | Food | Membrane | HMDB0039057 | met15880 |
|  | N-Desmethyleletriptan | 368.155849 | C21H24N2O2S | Organic compounds | Drug metabolite\|Endogenous | Extracellular | HMDB0013919 | met72250 |
|  | 2,4-Dihydroxy-4,6-dimethoxy-3-prenylchalcone | 368.162374 | C22H24O5 | Organic compounds | Food | Membrane | HMDB0038815 | met57650 |
|  | Dehydroepiandrosterone sulfate | 368.165745 | C19H28O5S | Lipids and lipid-like molecules | Drug or steroid metabolite\|Endogenous\|Food | Cytoplasm\|Extracellular\|Membrane | HMDB0001032 | met32021 |
|  | Testosterone sulfate | 368.165745 | C19H28O5S | Organic compounds | Drug or steroid metabolite\|Endogenous\|Food | Cytoplasm\|Extracellular\|Membrane (predicted from logP) | HMDB0002833 | met21389 |
| **2423** | Licarin C | 370.178024 | C22H26O5 | Organic compounds | Food | Membrane | HMDB0033319 | met70031 |
|  | Kanzonol R | 370.178024 | C22H26O5 | Organic compounds | Food | Membrane | HMDB0041099 | met15846 |
|  | Androsterone sulfate | 370.181395 | C19H30O5S | Organic compounds | Drug or steroid metabolite\|Endogenous\|Food | Cytoplasm\|Extracellular\|Membrane (predicted from logP)\|Endoplasmic reticulum | HMDB0002759 | met7567 |
|  | 5a-Dihydrotestosterone sulfate | 370.181395 | C19H30O5S | Organic compounds | Drug or steroid metabolite\|Endogenous\|Food | Cytoplasm\|Extracellular\|Membrane | HMDB0006278 | met28175 |
|  | Etiocholanolone sulfate | 370.181395 | C19H30O5S | Organic compounds | Endogenous\|Food | Extracellular\|Membrane | HMDB0013232 | met31426 |
| **192** | LysoPE(0:0/20:5(5Z,8Z,11Z,14Z,17Z)) | 499.269889 | C25H42NO7P | Organic compounds | Endogenous\|Food | Extracellular\|Membrane | HMDB0011489 | met54563 |
|  | LysoPE(20:5(5Z,8Z,11Z,14Z,17Z)/0:0) | 499.269889 | C25H42NO7P | Organic compounds | Endogenous\|Food | Extracellular\|Membrane | HMDB0011519 | met61017 |
| **335** | LysoPE(0:0/22:6(4Z,7Z,10Z,13Z,16Z,19Z)) | 525.285539 | C27H44NO7P | Organic compounds | Endogenous\|Food | Extracellular\|Membrane | HMDB0011496 | met40479 |
|  | LysoPE(22:6(4Z,7Z,10Z,13Z,16Z,19Z)/0:0) | 525.285539 | C27H44NO7P | Organic compounds | Endogenous\|Food | Extracellular\|Membrane | HMDB0011526 | met10259 |
| **1404** | LysoPE(0:0/24:6(6Z,9Z,12Z,15Z,18Z,21Z)) | 553.316839 | C29H48NO7P | Organic compounds | Endogenous\|Food | Extracellular\|Membrane | HMDB0011499 | met63747 |
|  | LysoPE(24:6(6Z,9Z,12Z,15Z,18Z,21Z)/0:0) | 553.316839 | C29H48NO7P | Organic compounds | Endogenous\|Food | Extracellular\|Membrane | HMDB0011529 | met33507 |
| **606** | LysoPC(18:0) | 523.36379 | C26H54NO7P | Organic compounds | Endogenous\|Food | Extracellular\|Membrane | HMDB0010384 | met29482 |
|  | LysoPC(0:0/18:0) | 523.36379 | C26H54NO7P | Organic compounds | Endogenous\|Food | Extracellular\|Membrane | HMDB0011128 | met47958 |
| **3565** | Histamine | 111.079647 | C5H9N3 | Organic nitrogen compounds | Endogenous | Extracellular | HMDB0000870 | met46141 |
| **86/87/1036** | Spermine | 202.215747 | C10H26N4 | Organic compounds | Endogenous | Cytoplasm | HMDB0001256 | met8665 |
| **949** | 2,4-Dihydroxy-7-methoxy-2H-1,4-benzoxazin-3(4H)-one | 211.048072 | C9H9NO5 | Organoheterocyclic compounds | Drug\|Food | Cytoplasm\|Extracellular | HMDB0034864 | met74038 |
|  | Betalamic acid | 211.048072 | C9H9NO5 | Organic acids and derivatives | Endogenous\|Food | Cytoplasm\|Extracellular | HMDB0029842 | met2485 |
|  | Phosphocreatine | 211.035807 | C4H10N3O5P | Organic acids and derivatives | Endogenous | Mitochondria | HMDB0001511 | met66385 |
|  | Topaquinone | 211.048072 | C9H9NO5 | Organic compounds | Endogenous | NULL | HMDB0011639 | met34259 |
| **326/638** | D-Fructose | 180.063388 | C6H12O6 | Organic oxygen compounds | Endogenous | Extracellular | HMDB0000660 | met52449 |
|  | Methionine sulfoximine | 180.056863 | C5H12N2O3S | Organic acids and derivatives | Endogenous\|Food | Cytoplasm\|Extracellular | HMDB0029430 | met2484 |
|  | Allose | 180.063388 | C6H12O6 | Organic compounds | Endogenous | Cytoplasm (predicted from logP) | HMDB0001151 | met36255 |
|  | L-Sorbose | 180.063388 | C6H12O6 | Organic compounds | Endogenous\|Microbial | Cytoplasm | HMDB0001266 | met55699 |
|  | Beta-D-Galactose | 180.063388 | C6H12O6 | Organic oxygen compounds | Endogenous | Extracellular\|Lysosome | HMDB0003449 | met57655 |
|  | Myoinositol | 180.063388 | C6H12O6 | Organic oxygen compounds | Endogenous | Extracellular | HMDB0000211 | met62238 |
|  | D-Galactose | 180.063388 | C6H12O6 | Organic oxygen compounds | Endogenous | Extracellular\|Lysosome | HMDB0000143 | met37272 |
|  | D-Mannose | 180.063388 | C6H12O6 | Organic compounds | Endogenous | Cytoplasm\|Extracellular\|Lysosome\|Endoplasmic reticulum\|Golgi apparatus | HMDB0000169 | met24133 |
|  | Isonicotinylglycine | 180.053492 | C8H8N2O3 | Organic compounds | Drug metabolite\|Endogenous | NULL | HMDB0041912 | met3166 |
|  | Theophylline | 180.064726 | C7H8N4O2 | Organic compounds | Drug metabolite\|Food | Cytoplasm (predicted from logP) | HMDB0001889 | met60855 |
|  | Levoinositol | 180.063388 | C6H12O6 | Organic compounds | Food | Cytoplasm\|Extracellular | HMDB0034220 | met58441 |
|  | L-Galactose | 180.063388 | C6H12O6 | Organic compounds | Food | Cytoplasm\|Extracellular | HMDB0033704 | met70708 |
|  | 3-Deoxyarabinohexonic acid | 180.063388 | C6H12O6 | Organic compounds | Endogenous | Cytoplasm (predicted from logP) | HMDB0000346 | met38592 |
|  | D-Glucose | 180.063388 | C6H12O6 | Organic compounds | Endogenous | Extracellular\|Lysosome\|Endoplasmic reticulum\|Golgi apparatus | HMDB0000122 | met71048 |
|  | Alpha-D-Glucose | 180.063388 | C6H12O6 | Organic oxygen compounds | Endogenous | Cytoplasm (predicted from logP) | HMDB0003345 | met45789 |
|  | 12-Tridecene-4,6,8,10-tetraynal | 180.057515 | C13H8O | Organic compounds | Endogenous\|Food | Extracellular\|Membrane | HMDB0038915 | met67276 |
|  | D-Tagatose | 180.063388 | C6H12O6 | Organic compounds | Endogenous | NULL | HMDB0003418 | met68898 |
|  | Beta-D-Glucose | 180.063388 | C6H12O6 | Organic oxygen compounds | Endogenous | Cytoplasm | HMDB0000516 | met18467 |
|  | Dihydroxyacetone (dimer) | 180.063388 | C6H12O6 | Organoheterocyclic compounds | Endogenous\|Food | Cytoplasm\|Extracellular | HMDB0032222 | met7024 |
|  | L-Gulose | 180.063388 | C6H12O6 | Organic compounds | Food\|Microbial\|Plant | NULL | HMDB0012326 | met4783 |
|  | Nicotinuric acid | 180.053492 | C8H8N2O3 | Organic compounds | Endogenous | Cytoplasm (predicted from logP) | HMDB0003269 | met58442 |
|  | Adrenochrome o-semiquinone | 180.066068 | C9H10NO3 | Organic compounds | Endogenous | NULL | HMDB0012883 | met48439 |
| **4054** | Valproylglycine | 201.136494 | C10H19NO3 | Organic compounds | Endogenous | NULL | HMDB0013116 | met13313 |
|  | Capryloylglycine | 201.136494 | C10H19NO3 | Organic compounds | Endogenous | Membrane | HMDB0000832 | met14777 |
|  | N-(5-Methyl-3-oxohexyl)alanine | 201.136494 | C10H19NO3 | Organic compounds | Food | Cytoplasm\|Extracellular | HMDB0041540 | met58824 |
| **1156** | Galactonic acid | 196.058303 | C6H12O7 | Organic compounds | Endogenous | Cytoplasm | HMDB0000565 | met31894 |
|  | 3,7-Dimethyluric acid | 196.05964 | C7H8N4O3 | Organic compounds | Drug metabolite\|Endogenous | Cytoplasm | HMDB0001982 | met19116 |
|  | Gluconic acid | 196.058303 | C6H12O7 | Organic oxygen compounds | Endogenous | Cytoplasm (predicted from logP) | HMDB0000625 | met8923 |
|  | 7,9-Dimethyluric acid | 196.05964 | C7H8N4O3 | Organic compounds | Drug metabolite\|Endogenous | Cytoplasm | HMDB0004308 | met61674 |
|  | Gulonic acid | 196.058303 | C6H12O7 | Organic compounds | Endogenous | Endoplasmic reticulum | HMDB0003290 | met27231 |
|  | 1,9-Dimethyluric acid | 196.05964 | C7H8N4O3 | Organic compounds | Drug metabolite\|Endogenous | Cytoplasm | HMDB0002026 | met5608 |
|  | 1,3-Dimethyluric acid | 196.05964 | C7H8N4O3 | Organic compounds | Drug metabolite\|Endogenous | Cytoplasm (predicted from logP) | HMDB0001857 | met37884 |
|  | Ethyl 1-(ethylthio)propyl disulfide | 196.041413 | C7H16S3 | Organic compounds | Food | Membrane | HMDB0033045 | met25205 |
|  | 1-(Methylthio)propyl propyl disulfide | 196.041413 | C7H16S3 | Organosulfur compounds | Food | Membrane | HMDB0033044 | met3107 |
|  | 1,7-Dimethyluric acid | 196.05964 | C7H8N4O3 | Organic compounds | Drug metabolite\|Endogenous | Cytoplasm | HMDB0011103 | met62304 |
| **1894** | Gamma-delta-Dioxovaleric acid | 130.026609 | C5H6O4 | Organic compounds | Endogenous | NULL | HMDB0013233 | met16283 |
|  | Mesaconic acid | 130.026609 | C5H6O4 | Organic compounds | Endogenous\|Food | Extracellular\|Membrane\|Mitochondria | HMDB0000749 | met27937 |
|  | Citraconic acid | 130.026609 | C5H6O4 | Lipids and lipid-like molecules | Endogenous\|Food | Extracellular\|Membrane | HMDB0000634 | met10299 |
|  | Methyl hydrogen fumarate | 130.026609 | C5H6O4 | Lipids and lipid-like molecules | Endogenous\|Food | Cytoplasm\|Extracellular\|Membrane | HMDB0033809 | met33665 |
|  | Itaconic acid | 130.026609 | C5H6O4 | Lipids and lipid-like molecules | Endogenous | Mitochondria | HMDB0002092 | met35628 |
|  | Glutaconic acid | 130.026609 | C5H6O4 | Organic acids and derivatives | Endogenous\|Food | Extracellular\|Membrane | HMDB0000620 | met46852 |
| **1923** | 1-(2H-1,3-Benzodioxol-5-yl)-2-[2,6-dimethoxy-4-(prop-2-en-1-yl)phenoxy]propyl benzoate | 476.183503 | C28H28O7 | Organic compounds | Food | Membrane | HMDB0039249 | met8770 |
| **1930** | Riboflavin reduced | 348.106984 | C15H16N4O6 | Organic compounds | Endogenous | Cytoplasm (predicted from logP) | HMDB0001557 | met50446 |
| **1122** | Desglucocoroloside | 504.308704 | C29H44O7 | Organic compounds | Endogenous\|Food | Cytoplasm\|Extracellular\|Membrane | HMDB0033709 | met7794 |
| **2644** | Pyrrolidine | 71.0734993 | C4H9N | Organoheterocyclic compounds | Endogenous\|Food\|Microbial | Cytoplasm\|Extracellular | HMDB0031641 | met27175 |
| **4223** | Queuosine | 409.159748 | C17H23N5O7 | Organic compounds | Endogenous | NULL | HMDB0011596 | met48977 |
| **4322** | Dihydrothymine | 128.058578 | C5H8N2O2 | Organic compounds | Endogenous | Cytoplasm | HMDB0000079 | met5187 |
|  | xi-5-Acetyltetrahydro-2(3H)-furanone | 128.047344 | C6H8O3 | Organic compounds | Food | Cytoplasm\|Extracellular | HMDB0038072 | met42387 |
|  | 3-Hydroxy-4,5-dimethyl-2(5H)-furanone | 128.047344 | C6H8O3 | Organoheterocyclic compounds | Endogenous\|Food | Cytoplasm\|Extracellular | HMDB0031306 | met67167 |
|  | L-erythro-5-(1-Hydroxyethyl)-2(5H)-furanone | 128.047344 | C6H8O3 | Organic compounds | Food | Cytoplasm\|Extracellular | HMDB0033285 | met13440 |
|  | L-Cyclo(alanylglycyl) | 128.058578 | C5H8N2O2 | Organoheterocyclic compounds | Endogenous\|Food | Cytoplasm\|Extracellular | HMDB0031547 | met28043 |
|  | Squamolone | 128.058578 | C5H8N2O2 | Organic compounds | Endogenous\|Food | Cytoplasm\|Extracellular | HMDB0029874 | met70129 |
|  | 2,7-Oxepanedione | 128.047344 | C6H8O3 | Organoheterocyclic compounds | Food | Cytoplasm\|Extracellular | HMDB0040732 | met25773 |
|  | (¬±)-Furaneol | 128.047344 | C6H8O3 | Organic compounds | Food | Cytoplasm\|Extracellular | HMDB0040594 | met50681 |
|  | 2-Ethyl-3,4-dihydroxyfuran | 128.047344 | C6H8O3 | Organoheterocyclic compounds | Endogenous\|Food | NULL | HMDB0031847 | met22377 |
|  | Osmundalactone | 128.047344 | C6H8O3 | Organic compounds | Endogenous\|Food | Cytoplasm\|Extracellular | HMDB0031303 | met25900 |
|  | 5,6-Dihydro-4-methoxy-2H-pyran-2-one | 128.047344 | C6H8O3 | Organoheterocyclic compounds | Food\|Toxin | Cytoplasm\|Extracellular | HMDB0033517 | met14798 |
| **1162** | 2-Methylene-4-oxopentanedioic acid | 158.021523 | C6H6O5 | Organic compounds | Food | Cytoplasm\|Extracellular | HMDB0037759 | met9529 |
|  | Zymonic acid | 158.021523 | C6H6O5 | Organoheterocyclic compounds | Endogenous\|Food | Cytoplasm\|Extracellular | HMDB0031210 | met74508 |
| **471** | Ascladiol | 156.042259 | C7H8O4 | Organic compounds | Endogenous\|Food | Cytoplasm\|Extracellular | HMDB0029610 | met41589 |
|  | Imidazolelactic acid | 156.053492 | C6H8N2O3 | Organic compounds | Endogenous | Cytoplasm (predicted from logP) | HMDB0002320 | met13466 |
|  | 4-Imidazolone-5-propionic acid | 156.053492 | C6H8N2O3 | Organic compounds | Endogenous | Cytoplasm (predicted from logP) | HMDB0001014 | met71269 |
|  | 5-Hydroxymethyl-4-methyluracil | 156.053492 | C6H8N2O3 | Organic compounds | Endogenous | Cytoplasm | HMDB0000544 | met70518 |
| **598** | Histidinal | 139.074562 | C6H9N3O | Organic compounds | Endogenous | NULL | HMDB0012234 | met68527 |
|  | 5-Acetyl-2,4-dimethyloxazole | 139.063329 | C7H9NO2 | Organic compounds | Food | Cytoplasm\|Extracellular | HMDB0038174 | met67926 |
|  | 3,4-Dihydroxybenzylamine | 139.063329 | C7H9NO2 | Organic compounds | Endogenous | NULL | HMDB0012153 | met27307 |
| **2149** | Aniline | 93.0578492 | C6H7N | Benzenoids | Endogenous | NULL | HMDB0003012 | met40067 |
| **2204** | Ethyl N-methylanthranilate | 179.094629 | C10H13NO2 | Benzenoids | Food | Cytoplasm\|Extracellular | HMDB0033763 | met10279 |
|  | Salsolinol | 179.094629 | C10H13NO2 | Organic compounds | Endogenous | NULL | HMDB0042012 | met23504 |
|  | (R)-Salsolinol | 179.094629 | C10H13NO2 | Organic compounds | Endogenous | NULL | HMDB0005199 | met25789 |
|  | 1,2,3,4,5,6-Hexahydro-5-(1-hydroxyethylidene)-7H-cyclopenta[b]pyridin-7-one | 179.094629 | C10H13NO2 | Organic compounds | Endogenous\|Food | Cytoplasm\|Extracellular\|Membrane | HMDB0039655 | met18737 |
|  | Maltoxazine | 179.094629 | C10H13NO2 | Organoheterocyclic compounds | Endogenous\|Food | Cytoplasm\|Extracellular | HMDB0030372 | met30353 |
|  | 3,5-Dimethylphenyl methylcarbamate | 179.094629 | C10H13NO2 | Organic compounds | Endogenous\|Food | Cytoplasm\|Extracellular | HMDB0031811 | met29072 |
|  | 2(N)-Methyl-norsalsolinol | 179.094629 | C10H13NO2 | Organic compounds | Endogenous | NULL | HMDB0001189 | met34483 |
|  | 2,3-Dihydro-5-(3-hydroxypropanoyl)-1H-pyrrolizine | 179.094629 | C10H13NO2 | Organoheterocyclic compounds | Food | Cytoplasm\|Extracellular | HMDB0040021 | met14579 |
| **2940** | Oudemansin A | 290.151809 | C17H22O4 | Benzenoids | Food | Membrane | HMDB0039949 | met73494 |
|  | Laurenobiolide | 290.151809 | C17H22O4 | Lipids and lipid-like molecules | Endogenous\|Food | Extracellular\|Membrane | HMDB0036771 | met32326 |
|  | [6]-Dehydrogingerdione | 290.151809 | C17H22O4 | Phenylpropanoids and polyketides | Endogenous\|Food | Membrane | HMDB0029474 | met74217 |
|  | Diisopentyl thiomalate | 290.15518 | C14H26O4S | Lipids and lipid-like molecules | Endogenous\|Food | Membrane | HMDB0032223 | met19833 |
| **3329** | Ethyl N-methylanthranilate | 179.094629 | C10H13NO2 | Benzenoids | Food | Cytoplasm\|Extracellular | HMDB0033763 | met10279 |
|  | Salsolinol | 179.094629 | C10H13NO2 | Organic compounds | Endogenous | NULL | HMDB0042012 | met23504 |
|  | (R)-Salsolinol | 179.094629 | C10H13NO2 | Organic compounds | Endogenous | NULL | HMDB0005199 | met25789 |
|  | 1,2,3,4,5,6-Hexahydro-5-(1-hydroxyethylidene)-7H-cyclopenta[b]pyridin-7-one | 179.094629 | C10H13NO2 | Organic compounds | Endogenous\|Food | Cytoplasm\|Extracellular\|Membrane | HMDB0039655 | met18737 |
|  | Maltoxazine | 179.094629 | C10H13NO2 | Organoheterocyclic compounds | Endogenous\|Food | Cytoplasm\|Extracellular | HMDB0030372 | met30353 |
|  | 3,5-Dimethylphenyl methylcarbamate | 179.094629 | C10H13NO2 | Organic compounds | Endogenous\|Food | Cytoplasm\|Extracellular | HMDB0031811 | met29072 |
|  | 2(N)-Methyl-norsalsolinol | 179.094629 | C10H13NO2 | Organic compounds | Endogenous | NULL | HMDB0001189 | met34483 |
|  | 2,3-Dihydro-5-(3-hydroxypropanoyl)-1H-pyrrolizine | 179.094629 | C10H13NO2 | Organoheterocyclic compounds | Food | Cytoplasm\|Extracellular | HMDB0040021 | met14579 |
| **3965** | 3-Methyl-9H-carbazole-9-carboxaldehyde | 209.084064 | C14H11NO | Organic compounds | Food\|Plant | Membrane | HMDB0032757 | met54040 |
|  | 10-Methylacridone | 209.084064 | C14H11NO | Organic compounds | Food | Membrane | HMDB0033000 | met73385 |
| **4391** | (R,S)-Norlaudanosoline | 287.115758 | C16H17NO4 | Organic compounds | Endogenous | NULL | HMDB0012486 | met6493 |
|  | Pipermethystine | 287.115758 | C16H17NO4 | Organic compounds | Food | Cytoplasm\|Extracellular | HMDB0033486 | met1178 |
| **3594** | Unknown | 312.1545 | C12 H20 N6 O4 | Unknown | Unknown | Unknown | Unknown | Unknown |
| **4387** | Unknown | 265.15222 | C11 H23 N O6 | Unknown | Unknown | Unknown | Unknown | Unknown |
| **2427** | Unknown | 306.05205 | C14 H16 N2 P2 S | Unknown | Unknown | Unknown | Unknown | Unknown |
| **808** | Unknown | 587.32255 |  | Unknown | Unknown | Unknown | Unknown | Unknown |
| **943** | Unknown | 613.33828 | C38 H47 N O6 | Unknown | Unknown | Unknown | Unknown | Unknown |
| **1209** | Unknown | 609.32544 |  | Unknown | Unknown | Unknown | Unknown | Unknown |
| **1366** | Unknown | 681.32566 | C36 H47 N3 O10 | Unknown | Unknown | Unknown | Unknown | Unknown |
| **1386** | Unknown | 595.28814 | C26 H42 N7 O7 P | Unknown | Unknown | Unknown | Unknown | Unknown |
| **1393** | Unknown | 610.26271 | C25 H39 N8 O8 P | Unknown | Unknown | Unknown | Unknown | Unknown |
